# Association Between Hypertension and Hearing Loss

**DOI:** 10.7759/cureus.18025

**Published:** 2021-09-16

**Authors:** Muhammad Umair Nawaz, Sagar Vinayak, Edgar Rivera, Kanwal Elahi, Hamza Tahir, Vishal Ahuja, Sana Jogezai, Waseem Maher, Sidra Naz

**Affiliations:** 1 Internal Medicine, Jinnah Sindh Medical University, Karachi, PAK; 2 Internal Medicine, American University of Barbados, Bridgetown, BRB; 3 Internal Medicine, Merchant Logo Universidad Autónoma de Guadalajara, Zapopan, MEX; 4 Internal Medicine, Allama Iqbal Medical College, Lahore, PAK; 5 Internal Medicine, University of Health Sciences, Lahore, PAK

**Keywords:** sensorineural hearing loss, hypertension, hearing loss, htn, ototoxic drugs

## Abstract

Introduction

Hypertension (HTN) is a common health problem, diagnosed in every one out of four individuals. It is associated with various complications; however, its impact on hearing loss is not well studied. In this study, we will determine the impact of HTN on hearing.

Methods

This cross-sectional study was conducted in Jinnah Sindh Medical University from August 2020 to March 2021. Three hundred (300) patients with documented diagnosis of HTN, between the ages of 21 and 50 years, were enrolled in the study. Another 300 non-hypertensive participants were enrolled as a reference group. Participants were sent to trained otolaryngologist technicians, who performed audiometry at six different frequencies for each year (0.5, 1.0, 2.0, 3.0, 4.0, and 6.0 kilohertz (kHz)). The final hearing level was calculated by taking mean of hearing levels of both ears.

Results

The hearing levels in audiometry were significantly higher in hypertensive participants compared to non-hypertensive participants (23.4 ± 8.67 dB vs 18.3 ± 6.02 dB; p-value: <0.0001). Participants who had been diagnosed with HTN for more than five years had higher hearing levels in audiometry test compared to participants with less than five years of HTN (24.21 ± 8.92 dB vs. 22.6 ± 8.02 dB; p-value 0.0001).

Conclusion

Based on our study, HTN is positively correlated with hearing loss. Therefore, longstanding hypertensive patients should be screened regularly in order to assess the status of their hearing abilities.

## Introduction

Hypertension (HTN), a global health issue, has been observed in one out of four adults. On estimation, approximately 3.5 billion adults are reported to show systolic blood pressure (SBP) of >110-115 mmHg, which is considered to be non-optimal [1]. On the other hand, SBP readings of >140 mmHg are seen in up to 874 million adults, which is not normal. A study suggested that non-optimal SBP readings are considered to be an important parameter in assessing HTN, leading to approximately 9.4 million deaths annually [1]. Another study conducted in 2010 with similar objectives reported approximately 7.5 million deaths, which makes up around 12.8% of the yearly deaths, globally [2].

One of the complications associated with HTN is sensorineural hearing loss (HL). Literature provides evidence supporting the fact that HTN could potentially lead to HL [3]. The possible explanation for this could be the reduced blood circulation to the cochlea. Decreased blood circulation, in turn, causes decreased oxygen supply to the cochlea, increased formation of free radicals, increased loss of ear cells, and disturbed recycling of the ions. Therefore, all these factors could cause a malfunction of the inner ear, resulting in HL and tinnitus [4]. In concordance with this fact, a study suggested that hypertensive patients were reported to have higher hearing thresholds as well as an increased prevalence of tinnitus [5]. Another study assessing the association between HL and HTN examined hearing by using frequencies between 250 and 8000 Hz to measure the pure tone threshold. It was concluded that HTN and HL have a positive correlation. Age was also reported to be a major risk factor in hypertensive patients to cause HL [6].

While a lot of data are available related to complications of HTN, very little work has been done to investigate the effect of HTN on hearing. In this case-control study, we will determine the impact of HTN on hearing.

## Materials and methods

This cross-sectional study was conducted in Jinnah Sindh Medical University from August 2020 to March 2021. Three hundred (300) patients with documented diagnosis of HTN, between the ages of 21 and 50 years, were enrolled in the study. Ethical review board approval was taken from the institute (JSMU/IRB-OfC/2020-05-07). These patients were selected from out patient department, who came for their follow-up. Three hundred participants matched for age and gender were also enrolled in the study as control group. These control group were attendant and/or relatives of case group, who accompanied the case group. These participants were included in the study using consecutive convenient non-probability sampling. Each participant was explained the study protocol and their consent was taken.

After enrollment, demographics and clinical values of participants were noted in self-structured questionnaire. These included age of participants, body mass index (BMI), diastolic blood pressure (DBP), and SBP. After noting these characteristics, participants were sent to the same trained otolaryngologist technician. He performed audiometry at six different frequencies for each year (0.5, 1.0, 2.0, 3.0, 4.0, and 6.0 kilohertz (kHz)). The final hearing level was calculated by taking mean of hearing levels of both ears. 

The data were statistically analyzed using Statistical Package for the Social Sciences, v. 23.0 (SPSS, IBM Corporation, Armonk, New York, United States). Categorical data such as age distribution were presented as frequency and percentage. Numerical data such as BMI and hearing levels were presented as mean and standard deviation. T-test and chi-square test were applied as appropriate. A p-value of less than 0.05 meant that the difference between the groups is significant and the null hypothesis is void.

## Results

The distribution of age group, BMI, and DBP was comparable between the two groups. However, the SBP was significantly higher in smokers compared to non-smokers (119.12 ± 9.2 mmHg vs. 113.12 ± 8.7 mmHg; p-value: <0.0001) (Table 1).

**Table 1:** Comparison of characteristics of both groups BMI: body mass index, DBP: diastolic blood pressure, kg/m2: kilograms per square meter, mmHg: millimeters of mercury, NS: nonsignificant, SBP: systolic blood pressure

Characteristics	Hypertensive participants (n=300)	Non-hypertensive participants (n=300)	p-Value
Age group (in years)			
21-30	32 (10.6%)	30 (10.0%)	NS
31-40	69 (23.0%)	65 (21.6%)	
41-50	199 (66.3%)	205 (68.3%)	
Gender			
Male	181 (60.3%)	178 (59.3%)	NS
Female	119 (39.6%)	122 (40.6%)	
Measurements			
BMI (kg/m^2^)	23.8 ± 3.1	23.1 ± 3.3	NS
SBP (mmHg)	125.12 ± 10.6	111.81 ± 8.5	<0.0001
DBP (mmHg)	86.76 ± 7.2	73.31 ± 6.0	<0.0001

The hearing levels in audiometry were significantly higher in hypertensive participants compared to non-hypertensive participants (23.4 ± 8.67 dB vs 18.3 ± 6.02 dB; p-value: <0.0001) (Table 2).

**Table 2:** Hearing levels in hypertensive and non-hypertensive participants dB: decibels

Hearing levels (dB)	Hypertensive participants (n=300)	Non-hypertensive participants (n=300)	p-Value
	23.4 ± 8.67	18.3 ± 6.02	<0.0001

The duration of HTN also affected the hearing levels. Participants who had been diagnosed with HTN for more than five years had higher hearing levels in audiometry tests compared to participants with less than five years of HTN history (24.21 ± 8.92 dB vs. 22.6 ± 8.02 dB; p-value: 0.0001) (Table 3).

**Table 3:** Duration of smoking in hypertensive participants dB: decibels

Duration of smoking	Frequency (%)	Hearing levels (dB)	p-Value
Less than 5 years	167 (55.66%)	24.1 ± 8.92	<0.0001
More than 5 years	133 (44.33%)	22.6 ± 8.02	

## Discussion

Our study indicates that hypertensive patients were reported to have a considerably higher hearing level in audiometry than the non-hypertensive participants. When hypertensive patients were compared, those who had been hypertensive for more than five years had higher HL as compared to the non-hypertensive patients.

Literature providing evidence for the results of our study dates back to the early 20th century when the harmful effects of HTN on cochlear and vestibular systems in humans were hypothesized [7-9]. Ever since many studies on similar topics have been carried out with inconsistent outcomes. Baraldi et al. [10] conducted a study including aged people, which concluded that the extent of HL was more or less the same in people with and without HTN. However, the audiometric results were different in both groups. Another study by Agarwal et al. [6] included 150 hypertensive participants and 124 normotensive participants; hearing in both groups was compared. It was found that hypertensive patients with blood pressure over 180/110 mmHg were observed to bear worse hearing thresholds in high frequencies. Gates et al. [11] also found similar results in their study. Additionally, a relationship between HTN and low-frequency HL was found in females in these studies. On the contrary, other studies could not conclude a positive correlation between HTN and HL [10,12].

The possible explanation for the association between HL and HTN is thoroughly explained in several studies. The lateral wall of the cochlea has stria vascularis, which is meant to deliver auditory signals from the cochlea to the central nervous system [13]. The vessels that supply to the stria vascularis originate from the terminal arteries, with no support from collateral circulation. This is why it is extremely sensitive when the vascular supply is restricted; this is further confirmed by animal studies showing decreased endocochlear potential and HL right after an event causing decreased oxygen supply [14]. It is believed that HTN may cause restrictions in the vascular supply to the stria vascularis, potentially leading to HL [15]. Another possible explanation may be use of ototoxic anti-hypertensive medications such as loop diuretics. Loop diuretics were associated with the 10-year incidence of HL [16].

In light of the aforementioned findings, our study suggests that the use of ototoxic drugs in the management of HTN should be avoided since it puts patients at a higher risk of impaired hearing. Regular screening should be conducted in patients with HTN for early detection of HL. The study has its limitations as well. First, since the study was conducted in a single institute, sample size and diversity were limited. Secondly, since it was a cross-sectional study, a definite causative relationship between HTN and HL could not be established. Moreover, further multi-centered studies with a larger sample size and diversity are required to confirm the results.

## Conclusions

We speculate that HTN is positively correlated with HL. Therefore, longstanding hypertensive patients should be screened regularly in order to assess the status of their hearing abilities. In the case of pharmacological management of HTN, ototoxic drugs should be avoided.
